# The melanoma-specific graded prognostic assessment does not adequately discriminate prognosis in a modern population with brain metastases from malignant melanoma

**DOI:** 10.1038/bjc.2015.357

**Published:** 2015-10-20

**Authors:** Anna Wilkins, Andrew Furness, Richard W Corbett, Adam Bloomfield, Nuria Porta, Stephen Morris, Zohra Ali, James Larkin, Kevin Harrington

**Affiliations:** 1 Melanoma Unit, The Royal Marsden NHS Foundation Trust, Fulham Road, London SW3 6JJ, UK; 2 The Institute of Cancer Research, Division of Radiotherapy and Imaging, 123 Old Brompton Road, London SW7 3RP, UK; 3 The Institute of Cancer Research, Division of Clinical Studies, 123 Old Brompton Road, London SW7 3RP, UK; 4 Department of Medicine, Imperial College London, Hammersmith Hospital, Du Cane Road, London W12 0HS, UK; 5 Department of Clinical Oncology, Guy's and St Thomas' NHS Foundation Trust, Westminster Bridge Road, London SE1 7EH, UK

**Keywords:** brain metastases, melanoma, radiotherapy, graded prognostic assessment

## Abstract

**Background:**

The melanoma-specific graded prognostic assessment (msGPA) assigns patients with brain metastases from malignant melanoma to 1 of 4 prognostic groups. It was largely derived using clinical data from patients treated in the era that preceded the development of newer therapies such as BRAF, MEK and immune checkpoint inhibitors. Therefore, its current relevance to patients diagnosed with brain metastases from malignant melanoma is unclear. This study is an external validation of the msGPA in two temporally distinct British populations.

**Methods:**

Performance of the msGPA was assessed in Cohort I (1997–2008, *n*=231) and Cohort II (2008–2013, *n*=162) using Kaplan–Meier methods and Harrell's c-index of concordance. Cox regression was used to explore additional factors that may have prognostic relevance.

**Results:**

The msGPA does not perform well as a prognostic score outside of the derivation cohort, with suboptimal statistical calibration and discrimination, particularly in those patients with an intermediate prognosis. Extra-cerebral metastases, leptomeningeal disease, age and potential use of novel targeted agents after brain metastases are diagnosed, should be incorporated into future prognostic models.

**Conclusions:**

An improved prognostic score is required to underpin high-quality randomised controlled trials in an area with a wide disparity in clinical care.

Metastatic melanoma causes brain metastases in 30–60% of patients with stage IV disease (Fisher and Larkin, 2012). Treatment options include best supportive care (including corticosteroids), radiotherapy, neurosurgery, drug treatment or any combination of these. Radiotherapy may be delivered as whole brain radiotherapy (WBRT), stereotactic radiosurgery (SRS) or a combination of these treatments. Despite this range of available treatments, optimal management remains unclear and prognosis can vary considerably and unpredictably. Selecting patients appropriately for the most suitable treatment approach is extremely important to optimise outcomes, reduce toxicities and limit the overall financial impact of treatment. A number of scoring systems have been used, including a melanoma-specific RTOG recursive partitioning analysis (RPA) score (Morris *et al*, 2004).

More recently, the graded prognostic assessment (GPA) (Sperduto *et al*, 2008) has been used as a prognostic index for patients with brain metastases across tumour types. Similar to the RPA, this index is based on age, number of brain metastases, Karnofsky performance status (KPS) and the presence of extra-cranial metastases. The melanoma-specific GPA (msGPA) was derived from multivariate Cox regression assessment of these 4 factors in a North American cohort of 483 patients treated for brain metastases from melanoma between June 1993 and January 2010 (Sperduto *et al*, 2012). The msGPA is calculated from the sum of only two components, the KPS and the number of brain metastases (see Box 1). It may range from 0 to 4 and is straightforward to calculate at the time of diagnosis of intra-cerebral disease.

However, these scoring systems must be considered in the context of the changing landscape of melanoma treatment, resulting from the emergence of novel targeted agents. Inhibitors of BRAF kinase (vemurafenib, dabrafenib), MEK (trametinib, cobemetinib), and immune checkpoints (CTLA4 (ipilimumab) and PD-1 (pembrolizumab, nivolumab) have all emerged as active agents in melanoma and may have intra-cerebral activity in some patients (Fisher and Larkin, 2012; Long *et al*, 2012; Margolin *et al*, 2012; Dummer *et al*, 2014). The original derivation data set for the msGPA demonstrated good discrimination between prognostic groups using Kaplan–Meier (KM) methods (Sperduto *et al*, 2012). However, there was a selection bias towards fitter patients in that 76% of patients received SRS and no patient received best supportive care alone. Critically, the impact of predictive and prognostic biomarkers (e.g., BRAF/RAS mutation) and the use of newer targeted agents on survival were not explicitly considered in that study. Therefore, the current relevance of the msGPA to patients with brain metastases from malignant melanoma is uncertain.

In addition, the msGPA has not yet been validated robustly in the published literature. The study by Royston and Altman (2013) highlights the importance of validating prognostic indices with particular reference to a model's discrimination and calibration. Ideally, validation should occur in an external cohort that differs in time, investigators and location with that of the derivation data set (Royston and Altman, 2013). Therefore, the aim of this study was to validate the msGPA in two temporally distinct UK cohorts, including patients treated before and after the emergence of new, highly active anti-melanoma therapies.

## Materials and Methods

Electronic patient records for two separate cohorts of patients with brain metastases from metastatic melanoma were reviewed. Cohort I was obtained by searching radiotherapy records from 1 July 1997 to 31 July 2008, including all patients treated with radiotherapy, with or without surgery and drug treatment. Cohort II was obtained by screening clinic attendance records from 1 August 2008 until 1 August 2013 to identify patients who had brain metastases diagnosed after 1 August 2008. Cohort II included patients treated with novel targeted agents and best supportive care. This study was approved by our institutional review board (SE224).

Data extracted from electronic patient records included basic demographics (age and sex), date of diagnosis of brain metastases, number of brain metastases and KPS at the time of diagnosis of brain metastases. The presence or absence of leptomeningeal disease and the number of sites of extra-cerebral metastases were also recorded. Data on the number and type of treatments received for brain metastases were collected including neurosurgery, stereotactic radiotherapy and WBRT. Routine BRAF testing began at our institution in March 2010. Therefore, where available, BRAF mutation status was recorded. Details of treatment with novel targeted agents, started after the diagnosis of brain metastasis, were collected. Intra-cerebral radiological response according to RECIST criteria on the first scan to assess response to the novel agents was recorded. For patients receiving ipilimumab, later scans were checked for a possible delayed response. Finally, date of death or last clinical review was recorded.

The msGPA was calculated as described previously (Box 1) for all patients at the time of diagnosis of brain metastases. Patients without imaging reports including number of brain metastases (15 patients) or for whom it was not possible to identify KPS at the time of diagnosis of brain metastases (17 patients) were excluded. Patients without a date of death were censored at their last date of clinical follow-up for the survival analysis.

The Sperduto study does not report details of model coefficients, hazard ratios, indices of discrimination or a baseline survival function for the derivation cohort (Sperduto *et al*, 2012). This means that direct statistical comparison was limited to use of KM methods (Royston and Altman, 2013). KM survival curves were constructed for Cohort I and Cohort II and these were compared with the survival curves published for the derivation data set for the msGPA. Despite the lack of statistical parameters available for comparison in the derivation data set, the Cox proportional hazards model was used to assess differences in survival between different prognostic groups in the validation cohorts. In addition, Harrell's c-index of concordance was calculated for both cohorts. This is recommended as a measure of discrimination (Royston and Altman, 2013) in preference to testing for statistical significance between prognostic groups in validation studies. Harrell's c-index is defined as the proportion of all usable (non-censored) patient pairs in which the predictions and outcomes are concordant (Harrell *et al*, 1996). A value of 0.5 indicates no predictive discrimination and a value of 1.0 indicates perfect separation of patients with different outcomes. The c-index does not assess calibration.

To assess the impact of BRAF status and treatment with BRAF inhibitors on survival in Cohort II, KM survival curves were constructed for patients with BRAF wild-type *vs* BRAF mutant tumours. Patients with BRAF mutant tumours were split into those treated or not treated with a BRAF inhibitor after diagnosis of brain metastases. The log-rank test was used to formally compare survival between patients with BRAF wild-type tumours and patients with BRAF mutant tumours irrespective of subsequent treatment. The log-rank test was also used to compare survival of patients with BRAF mutant tumours who did or did not receive a BRAF inhibitor after diagnosis of brain metastases. Univariate and multivariate Cox regression analysis was used to assess the impact of novel targeted agents on survival, alongside previously identified prognostic factors (Broadbent *et al*, 2004; Morris *et al*, 2004; Hofmann *et al*, 2007). The proportional hazards assumption of the Cox model was tested using Schoenfeld residuals. Any factor that had a *P*-value<0.1 on univariate analysis was entered into the multivariate analysis. All statistical analysis was conducted using STATA v13.1 (StataCorp LP, College Station, TX, USA).

## Results

Overall 231 patients in Cohort I and 162 patients in Cohort II were eligible for this analysis following exclusion of 21 patients due to missing KPS (17 patients) or number of brain metastases (15 patients). Baseline characteristics of the two cohorts are shown in Table 1. Median survival in Cohort I was 3.6 months (95% CI 2.8–4.5 months) and in Cohort II was 4.4 months (95% CI 3.5–4.8 months). Patients in each of the different prognostic groups were well-represented in both cohorts.

**Figure 1:**
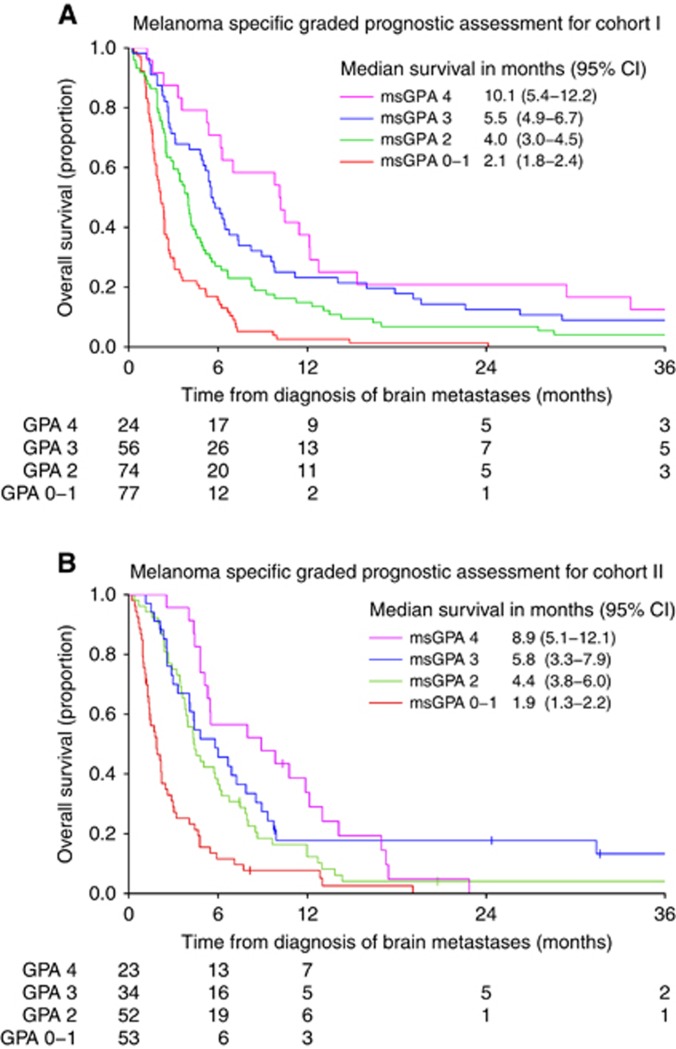
**Survival outcomes for patients with brain metastases from melanoma.** (**A** and **B**) Kaplan–Meier curves to show msGPA for Cohort I (1997–2008) and Cohort II (2008–2013). Abbreviation: msGPA=melanoma-specific graded prognostic assessment.

The KM curves shown in Figure 1 demonstrate that the msGPA enabled good discrimination between all four prognostic groups in Cohort I. However, in Cohort II, discrimination between groups was less good, particularly between patients with msGPA scores of 2 and 3. KM curves for msGPA scores 1 and 4 separate clearly from the rest of Cohort II, but the KM curves for msGPA scores 2 and 3 overlap for the first 6 months of follow-up and lie in close proximity for the remainder of the study period.

Harrell's c-index of concordance was 0.65 (95% CI 0.61–0.69) in Cohort I and 0.67 (95% CI 0.63–0.72) in Cohort II. A probability of concordance between observed and predicted outcomes of <0.7 indicates modest discrimination at best (Royston and Altman, 2013). Sperduto *et al* did not provide a c-index for their derivation cohort for comparison (Sperduto *et al*, 2012).

Median survival times for Cohorts 1 and 2 are shown in Table 2, together with median survival times from the Sperduto study (Sperduto *et al*, 2012). Median survival times for the Sperduto cohort differed considerably and were higher than both Cohorts I and II in all four msGPA groups, indicating poor calibration of the prognostic model. In GPA groups 3 and 4, median survivals were more than 3 months higher in the Sperduto cohort than in Cohorts I and II. For Cohorts I and II, the median survival times for the four different groups are ranked in the order predicted by the msGPA suggesting that, for this time point, discrimination of the model is reasonable. However, as discussed earlier, discrimination is less good in Cohort II at other time points. Hazard ratios for death for patients in the different msGPA groups for Cohorts I and II are also shown in Table 2. No data from Sperduto's derivation cohort are available for comparison, but, once again, the four different groups are ranked in the order predicted by the msGPA in both cohorts.

**Figure 2:**
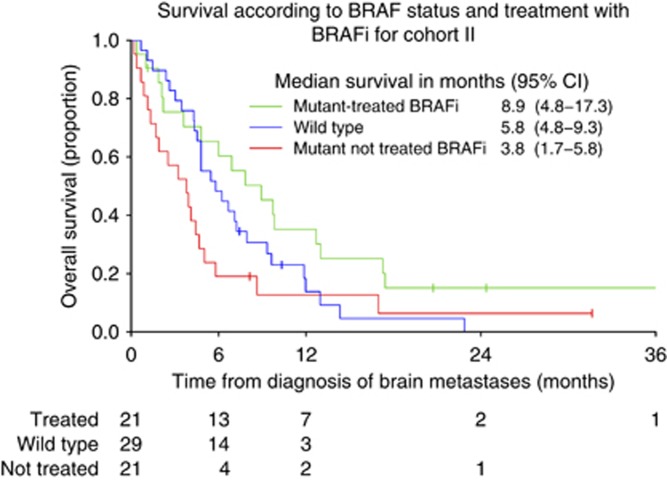
**Kaplan–Meier curves to show survival according to BRAF status and treatment with BRAF inhibitors (BRAFi) in Cohort II.**

BRAF mutation status was available for 71 patients in Cohort II—42 patients had a BRAF mutation, whereas tumours of 29 patients were BRAF wild-type. BRAF status was untested in 90 patients and unknown in 1 patient. The majority of untested patients were diagnosed with brain metastases between 2008 and 2010, when routine BRAF testing was not available at our institution. Survival of patients with BRAF wild-type tumours *vs* those with a BRAF mutation, divided into those who did or did not receive a BRAF inhibitor after diagnosis of brain metastases, is shown in Figure 2. There was no difference in survival between patients with BRAF mutation *vs* BRAF wild-type (log-rank test *P*=0.79). The KM curves, which are unadjusted for other prognostic factors, suggest that patients receiving a BRAF inhibitor after diagnosis of brain metastases had better survival outcomes than patients with BRAF mutant tumours who did not receive this treatment (log-rank test *P*=0.029).

Treatment with novel agents following diagnosis of brain metastases included vemurafenib (21 patients), ipilimumab (23 patients) or trametinib (1 patient). No patients received PD-1 antibodies. Intra-cerebral responses to the novel agents on the first radiological assessment of response are shown in Table 3. Overall 5 out of 45 patients experienced an intra-cerebral partial response to treatment, whereas 13 out of 45 patients experienced stable disease in the brain. No patients experienced a delayed response to ipilimumab in the brain.

Factors shown to predict survival on univariate analysis of patients in Cohort II included KPS, number of brain metastases, number of sites of extra-cerebral metastases, age and treatment with novel agents. Presence of leptomeningeal disease was of borderline significance (*P*=0.07). On multivariate analysis all five factors remained statistically significant predictors of survival (Table 4); once again leptomeningeal disease was of borderline significance (*P*=0.06).

## Discussion

To our knowledge, this study is the largest to date that seeks to validate the msGPA in a modern population. In the only other study in a cohort of 51 patients (Nieder *et al*, 2011), poor discrimination was reported between msGPA categories 0–1 and 2 using KM methods, and the authors concluded that the msGPA does not facilitate appropriate selection of patients for best supportive care. KPS and plasma lactate dehydrogenase levels (LDH) were identified as the only significant prognostic factors. In a slightly larger cohort of patients, the same authors found that only KPS and number of brain metastases significantly predicted prognosis (Nieder *et al*, 2012). A larger study of 251 patients treated with SRS concluded that the diagnosis-specific GPA splits patients into prognostically significant groups, but only 74 patients had melanoma and no melanoma-specific KM curves were reported. Therefore, the study is not informative about the performance of the msGPA (Likhacheva *et al*, 2012).

The population in this external validation study is representative of current UK practice. No baseline characteristics are available to describe the North American derivation cohort (Sperduto *et al*, 2012); however, the treatments given suggest that it differs considerably from the UK cohorts. For example, 16.7% of patients in Cohort II of this study received SRS, while 12.3% received best supportive care as compared with 76% and 0%, in Sperduto's derivation cohort (Sperduto *et al*, 2012). This is likely to be a significant factor explaining why the calibration between median survival outcomes in the derivation and validation data sets was poor. The difference in calibration is most marked in msGPA groups 3 and 4 and it is also possible that inferior therapies (e.g., less frequent use of SRS) in the UK resulted in inferior survival outcomes. As the number of systemic therapies that demonstrate intra-cerebral activity against metastatic melanoma increases (Long *et al*, 2012; Margolin *et al*, 2012; Dummer *et al*, 2014), the use of local treatments, such as SRS, for brain metastases is likely to expand, even in the UK.

Differences in the routine surveillance of metastatic melanoma between the UK and the US may be a further reason why calibration of the msGPA was poor. In the UK, surveillance for high risk stage III patients includes a brain scan, whereas for patients with stage IV disease brain scans are usually only carried out in response to neurological symptoms or prior to trial entry. In contrast, in the US, routine surveillance for stage IV patients often includes a brain scan. Brain metastases may, therefore, be detected earlier in the natural course of the disease when the patient has no or minimal symptoms and smaller metastases, meaning that aggressive local treatment may be more feasible and more likely to be effective.

While Cohort I and the original data set of Sperduto *et al* (2012) had good discriminatory ability between groups, the msGPA performed less well in Cohort II, particularly in patients with an intermediate prognosis (msGPA groups 2 and 3). The time frame and patients included in Cohort II are more relevant to current practice. In addition, patients with an intermediate prognosis are particularly in need of an efficient prognostic index to guide selection for locally aggressive treatments such as SRS or neurosurgery.

No significant differences in survival were seen between patients with wild-type *vs* mutant BRAF. Twenty-one of 42 patients (50%) with a BRAF mutation did not receive a BRAF inhibitor after diagnosis of brain metastases, usually because it had been given prior to development of radiologically visible brain metastases. Patients with BRAF mutant tumours who did not receive a BRAF inhibitor after the diagnosis of brain metastasis had a significantly worse prognosis than those who did receive such treatment. This finding is intriguing and certainly warrants further analysis in prospective studies of BRAF inhibition in the context of intra-cerebral disease.

Exposure to novel agents predicted for improved survival in the multivariate analysis, which accounted for KPS, number of brain metastases, leptomeningeal disease and extra-cerebral metastases. The intra-cerebral radiological responses seen suggest that such novel agents may exert their effects both inside and outside of the brain. A possible interpretation of these data is that patients with a future opportunity for targeted agents and newly diagnosed brain metastases might be particularly appropriate for more aggressive treatment of intra-cerebral disease. However, despite considerable progress in predicting which patients will respond to novel agents (Snyder *et al*, 2014; Tumeh *et al*, 2014), at present it remains difficult to reliably and reproducibly predict outcomes and further biomarkers of response are needed.

Other significant prognostic factors in the multivariate analysis that are not represented in the msGPA include the number of sites of extra-cerebral metastases and leptomeningeal disease. Both factors have been identified in previous studies, including a temporally distinct study at our institution (Morris *et al*, 2004). Age was also a significant prognostic factor, this is represented in the RPA classification of brain metastasis (Gaspar *et al*, 2000), which has been validated in patients with melanoma (Morris *et al*, 2004). Despite previous work suggesting female patients had better survival outcomes than male patients (Hofmann *et al*, 2007), our study did not show significant survival differences between men and women.

This study was a validation study that also explored new predictive factors relevant to current melanoma treatment. It did not aim to generate a new predictive model, which requires an independent data set (Royston and Altman, 2013) and a greater number of patients treated with a wider repertoire of novel targeted agents. The data presented in this study suggest that development of a new model incorporating factors unaccounted for in the msGPA would improve individualised treatment.

Two other recently proposed prognostic indices for patients with brain metastases treated with radiosurgery include the score index for radiosurgery (SIR) and the basic score for brain metastases (BSBM). The SIR includes age, KPS, systemic metastases, number of brain lesions and volume of lesions treated (Weltman *et al*, 2000). The BSBM is derived from KPS, control of treated brain lesions and presence or absence of extra-cranial disease (Lorenzoni *et al*, 2004). Of these, the BSBM has shown better survival prediction than the SIR and RPA (Lorenzoni *et al*, 2009), and has recently been updated to also predict neurological outcomes based on the addition of four brain factors (Serizawa *et al*, 2014). Neither index has been evaluated in a contemporary cohort of melanoma patients with brain metastases receiving novel targeted agents, but they do contain parameters that may prove important in risk stratification.

The retrospective nature of this analysis imposes some limitations on its interpretation. Cohort I included no patient receiving supportive measures alone and, thus, is unrepresentative of routine UK practice at that time. Meanwhile, Cohort II may have unintentionally excluded those patients with an accelerated clinical deterioration who were unable to attend outpatients for assessment or therapy. Despite recent data supporting LDH as an important prognostic factor in metastatic melanoma (Long *et al*, 2012; Larkin *et al*, 2014), it was not included as a prognostic factor because LDH was not measured routinely at our institution during the study period. Finally, the study included a simple validation method without detailed quantitative statistical parameters; however, these parameters were not published in the original study, precluding direct comparison.

In conclusion, this study externally validating the msGPA shows that this prognostic model has limited use in a modern population. In particular, it performs poorly for patients with an intermediate prognosis. Further work is needed to refine this model including incorporation of biomarkers (BRAF, RAS, CTLA4 and PD-L1/PD-1) and the effect of treatment options based on their presence or absence. Other prognostic factors previously shown to be important in this disease and excluded from the msGPA, including LDH (Staudt *et al*, 2010; Eigentler *et al*, 2011; Nieder *et al*, 2011; Partl *et al*, 2013), leptomeningeal disease (Morris *et al*, 2004), extra-cerebral metastases (Broadbent *et al*, 2004; Raizer, 2006; Rades *et al*, 2010) and size of intra-cranial metastases (Mathieu *et al*, 2007; Likhacheva *et al*, 2012), may also be relevant to future prognostic indices. Such indices are certainly needed to underpin high-quality randomised controlled trials in an area with a wide disparity in clinical care.

## Figures and Tables

**Table 1:** Basic demographics

	**Cohort I**	**Cohort II**
Total number of patients	231	162
Age, median (range)	55 (17–92)	58 (22–89)
**Sex,** ***n*** **(%)**		
Male	153 (66)	70 (43)
Female	78 (34)	92 (57)
**Number of brain metastases,** ***n*** **(%)**		
1	69 (30)	52 (32)
2–3	66 (29)	45(28)
>3	96 (41)	65 (40)
**KPS,** ***n*** **(%)**		
90–100	82 (36)	60 (37)
70–80	97 (42)	63 (39)
<70	52 (22)	39 (24)
**Extra-cerebral disease,** ***n*** **(%)**		
Yes	186 (81)	149 (92)
No	44 (19)	13 (8)
Not available	1	0
**Leptomeningeal disease,** ***n*** **(%)**		
Yes	7 (3)	8 (5)
No	223 (97)	150 (93)
Not available	1	4 (2)
**Treatment for brain metastases,** ***n*** **(%)**		
WBRT	226 (98)	104 (65)
SRS	24 (10)	27 (17)
Neurosurgery	39 (17)	26 (16)
Chemotherapy	67 (29)	31 (19)
Novel targeted agent^a^	0	41 (25)
Best supportive care	0	20 (12)

Abbreviation: KPS=Karnofsky performance status.

a Individual novel agents are outlined in Table 3.

**Table 2:** Hazard ratios of death for Cohorts I and II and median survival for Cohort I and II, and the derivation data set by Sperduto *et al*

	**Hazard ratio (95% CI)**^a^		**Median survival, months (95% CI)**		
**msGPA score**	**Cohort I**	**Cohort II**	**Cohort I**	**Cohort II**	**Sperduto** ***et al.***
0–1.0	1	1	2.1 (1.8–2.4)	1.9 (1.3–2.2)	3.38 (2.5–4.3)
2	0.5 (0.36–0.70)	0.45 (0.30–0.66)	4 (3.0–4.5)	4.4 (3.8–6.0)	4.7 (4.1–5.4)
3	0.34 (0.24–0.49)	0.31 (0.20–0.51)	5.5 (4.9–6.7)	5.8 (3.3–7.9)	8.8 (6.7–10.8)
4	0.25 (0.15–0.41)	0.28 (0.17–0.47)	10.1 (5.4–12.2)	8.9 (5.1–12.1)	13.2 (9.1–15.6)

Abbreviations: CI=confidence interval; msGPA=melanoma-specific graded prognostic assessment.

a Hazard ratios not reported by Sperduto *et al* (2012).

**Table 3:** BRAF status, novel agents and intra-cerebral response to novel agents in Cohort II

**BRAF status,** ***n*** **(%)**	***n*** **(%)**
Mutant	42 (25.9)
Wild type	29 (17.9)
Untested	90 (55.6)
BRAF status unknown	1 (0.6)
Total	162
**Novel agents used,** ***n*** **(%)**	
Vemurafenib	21 (46.7)
Ipilimumab	23 (51.1)
Trametinib	1 (2.2)
Total	45
**Intra-cerebral radiological response to vemurafenib,** ***n*** **(%)**	
Partial response	4 (19)
Stable disease	10 (47.6)
Progressive disease	2 (9.5)
Clinical progression, no scan	3 (14.2)
Not available	2 (9.5)
Total	21
**Intra-cerebral radiological response to ipilimumab,** ***n*** **(%)**	
Stable disease	3 (13.0)
Progressive disease	12 (52.2)
Clinical progression, no scan	5 (21.7)
Not available	3 (13.0)
Total	23
**Intra-cerebral radiological response to trametinib,** ***n*** **(%)**	
Partial response	1 (100%)

**Table 4:** Univariate and multivariate analysis of prognostic factors

	**Univariate analysis (*****n*****=162)**			**Multivariate analysis (*****n*****=138)**		
**Factor assessed**	**No.**	**Hazard ratio (95% CI)**	***P*****-value**	**No.**	**Hazard ratio (95% CI)**	***P*****-value**
**Karnofsky performance status**						
70	39	1		32	1	
70–80	63	0.55 (0.36–0.83)		53	0.42 (0.26–0.69)	
90–100	60	0.45 (0.30–0.69)	<0.001	53	0.35 (0.21–0.57)	<0.001
**Number of brain metastases**						
>3	65	1		51	1	
2–3	45	0.83 (0.56–1.23)		41	0.88 (0.53–1.44)	
1	52	0.41 (0.28–0.61)	<0.001	46	0.43 (0.27–0.70)	0.001
Age (continuous)	162	1.02 (1.01–1.03)	0.001	138	1.01 (0.99–1.02)	0.05
**Sex**						
Male	92	1				
Female	70	0.88 (0.64–1.22)	0.44	NA	NA	NA
**Sites of extra-cerebral metastases**						
Unknown	14			0		
4+	42	1		11	1	
3	36	0.60 (0.38–0.95)		25	0.64 (0.39–1.06)	
2	29	0.34 (0.20–0.56))		27	0.34 (0.20–0.61)	
1	30	0.31 (0.18–0.51)		34	0.29 (0.17–0.52)	
0	11	0.26 (0.12–0.56)	0.001	41	0.22 (0.10–0.50)	<0.001
**Leptomeningeal disease**						
Unknown	4			0		
No	150	1		132	1	
Yes	8	2.05 (0.94–4.44)	0.07	6	2.45 (0.95–6.33)	0.06
**Novel agents after diagnosis of BM**						
Unknown	11			0		
No	111	1		99	1	
Yes	40	0.42 (0.28–0.63)	<0.001	39	0.43 (0.28–0.66)	<0.001
**BRAF status**						
Unknown	91					
Wild type	29	1				
Mutant	42	0.93 (0.56–1.55)	0.79	NA	NA	NA

Abbreviations: BM=brain metastases; CI=confidence interval; NA=not assessed.

## Box 1.

Scoring the melanoma-specific graded prognostic assessment (msGPA)
